# Integration of tools for binding archetypes to SNOMED CT

**DOI:** 10.1186/1472-6947-8-S1-S7

**Published:** 2008-10-27

**Authors:** Erik Sundvall, Rahil Qamar, Mikael Nyström, Mattias Forss, Håkan Petersson, Daniel Karlsson, Hans Åhlfeldt, Alan Rector

**Affiliations:** 1 Department of Biomedical Engineering, Linköping University, 581 85 Linköping, Sweden; 2 School of Computer Science, University of Manchester, Kilburn Building, Oxford Road, Manchester, M13 9PL, UK

## Abstract

**Background:**

The Archetype formalism and the associated Archetype Definition Language have been proposed as an ISO standard for specifying models of components of electronic healthcare records as a means of achieving interoperability between clinical systems. This paper presents an archetype editor with support for manual or semi-automatic creation of bindings between archetypes and terminology systems.

**Methods:**

Lexical and semantic methods are applied in order to obtain automatic mapping suggestions. Information visualisation methods are also used to assist the user in exploration and selection of mappings.

**Results:**

An integrated tool for archetype authoring, semi-automatic SNOMED CT terminology binding assistance and terminology visualization was created and released as open source.

**Conclusion:**

Finding the right terms to bind is a difficult task but the effort to achieve terminology bindings may be reduced with the help of the described approach. The methods and tools presented are general, but here only bindings between SNOMED CT and archetypes based on the openEHR reference model are presented in detail.

## Background

Standardisation efforts in health informatics, including HL7, CEN, ISO, openEHR and IHTSDO, have provided EHR information model specifications as well as reference terminologies aiming at semantic interoperability [1]. Tools have been provided for managing the artefacts involved such as archetype editors (see ) and terminology browsers [2,3]. Yet, tools that support the integrated use of terminology and information models are not widespread.

This paper describes the integration of three applications related to archetypes and terminology systems,

a) an editor for archetype development,

b) MoST; a system for selecting terms from SNOMED CT to be bound to archetypes, and

c) TermViz; a tool for visualizing and navigating terminology systems.

The 'archetype' approach to information modelling is introduced below and is followed by descriptions of the three applications and their integration.

### Modelling in openEHR

The openEHR foundation  aims to facilitate interoperable implementations of electronic health record systems (EHRs), by developing and promoting open specifications and specifications-based implementations. The intention behind the specifications is to enable interoperability while still being flexible regarding information modelling design choices as well as choices of terminology systems, implementation technology, and human language translations.

The architecture of openEHR aims to scale from small desktop systems for general practitioners to distributed patient centred lifelong-shared care health record systems [4].

**Figure 1:**
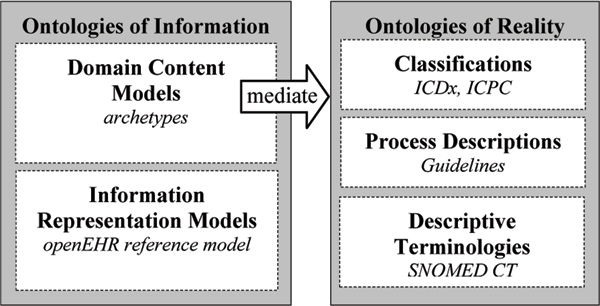
**Ontological structure**. Illustration of openEHR's ontological structure. Adapted from [1]

The openEHR architecture [4] includes a design principle called 'Ontological separation', which regulates the EHR modelling; see Figure 1. The structure is divided into two main categories entitled 'ontologies of information' and 'ontologies of reality'. Please note that the words 'Ontological' and 'ontologies' come from the source [4], but that in our opinion, 'models' could be equivalent.

The 'ontologies of information' contain the information models of the EHR content whereas the 'ontologies of reality' describe real phenomena with descriptions and classifications. The 'ontologies of information' are then divided into:

• 'Domain content models' containing formal definitions of the clinical content. They can be developed using archetypes, which are designed to be easy to change when new clinical needs arise. Detailed openEHR archetype information, examples and resources are available from 

• 'Information representation models' are implemented in the electronic health care systems software. They are used as a foundation for the domain content models and are designed to be stable with regards to model changes. In openEHR, this component is named the Reference model.

The 'ontologies of reality' contain e.g.:

• 'classifications', like ICDx and ICPC,

• 'process descriptions', like clinical guidelines,

• 'descriptive terminologies', like SNOMED CT.

EHR extracts based on common shared archetypes are proposed as a means to exchange information between different health care providers [4]. Semantics of the domain content models (e.g. archetypes) are provided by terminology binding. Meaning of nodes in archetypes is given by textual descriptions and optionally by reference to external terminology systems:

1. term definition – a node of an archetype is given meaning through a name and textual description,

2. term binding – a node of an archetype is given meaning by reference to an external terminology.

### SNOMED CT

SNOMED CT is the terminology system used for application in this paper. It is a clinical terminology based on concept representations that are related to each other by different types of relationships, like 'IS-A' (subtype), 'Part of', 'Causative agent' and many others. Each SNOMED CT *concept *representation is associated with a set of synonymous terms (coupled with metadata) called *descriptions *[5]. The number of active core concept representations in the January 2008 International Core release is 311 313. [6]

## Methods

The applications for archetype editing, semi-automatic terminology binding and terminology visualization that have been integrated are briefly described in this section.

### The archetype editor

Authoring of archetypes is not intended to be part of the daily routine of clinicians. Instead the goal is to develop archetypes that can be used in many different situations over a long period of time and to use them as parts of templates for clinical data entry.

**Figure 2:**
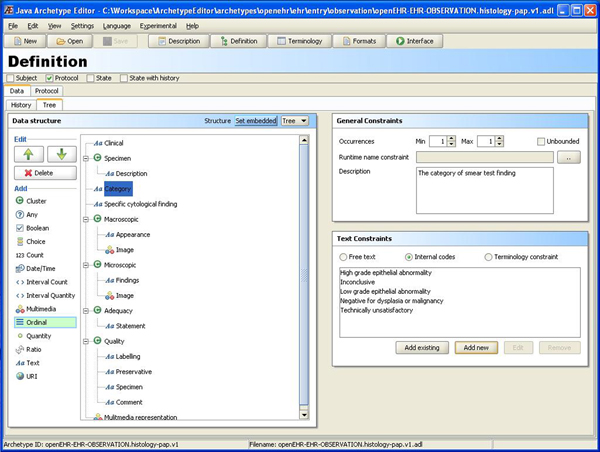
**Definition view**. The definition view of the archetype editor.

The purpose of the archetype editor is to let users build archetypes in an intuitive graphical environment, see Figure 2 without prior knowledge of formal representations of archetypes like the 'Archetype Definition Language' (ADL) or XML. We believe that an archetype editor that allows the user to create new archetypes and learn from previously created ones by viewing and exploring is important for developing good quality archetypes.

The development of the Java based archetype editor at Linköping University[7], was focused on improving terminology binding support and usability. In relation to already existing editors, it also removed operating system dependencies. Connections to external terminology sources like SNOMED CT and UMLS were included so that the effort required to bind terms with the help of external terminology sources was reduced compared to manual lookup.

### The MoST system

In order to bind nodes in clinical data models to nodes in external terminologies we must first find appropriate matches. The Model Standardisation using Terminology (MoST) system [8] developed at the University of Manchester is a general semi-automated mapping process providing the clinical modeller with candidate mappings. The mapping manually determined to be the most suitable can then be bound to a content model entity.

The specific clinical data models selected to demonstrate the applicability of the methodology in this paper are archetypes according to the openEHR archetype model, and SNOMED CT is the terminology to which they have been mapped to.

**Figure 3:**
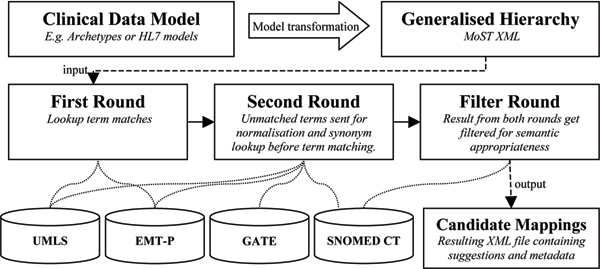
**MoST**. The system methodology of MoST.

In the MoST mapping process as shown in Figure 3, archetypes are converted from ADL format to a general XML format designed to represent hierarchical data models. The clinical content of the model is then passed to the actual mapping process, which executes various lexical and semanticprocedures by referring to existing medical resources (detailed below) and SNOMED CT.

The first round of mapping includes a lexical processing of terms using the Emergency Medical Text Processing (EMT-P) service. It is a natural language processing (NLP) tool, which cleans up raw text entries [9]. EMT-P then looks for matches in the Unified Medical Language System (UMLS) resources and the UMLS LVG database, which consists of normalised word forms (see, ).

The MoST methodology makes use of the lexical procedures of both the EMT-P tool and the UMLS resource at the same time to draw upon their individual and combined strengths to find relevant matches.

All archetype terms, irrespective of whether they have found a match in the first round, are sent to the second round for normalisation. Normalisation involves execution of a series of lexical and semantic methods and collation of results from each. Some of the methods employed include a training dataset with commonly used clinical synonyms and abbreviations, and context search. An external NLP application named GATE  was used for stemming, based on regular expression rules developed for its Morphological Analyzer, and synonym search using its WordNet  plugin.

At the end of both the rounds, the collated results are subjected to elimination through filtering. All filtered SNOMED CT results are presented to the clinical modeller as candidate mappings. The filtering and evaluation details are described in [8] as it is beyond the scope of this paper. Briefly, filtering comprises of two main levels. The first is exclusion of all concepts subsumed by a parent concept occurring in the result set, and inclusion of all non-occurring parent concepts if more than three child concepts are present in the result set. The second level involves inclusion of only those results whose semantic category (ies) is similar to the one specified by the clinical modeller. However, MoST provides for the possibility of a human and/or SNOMED CT categorisation error.

**Figure 4:**
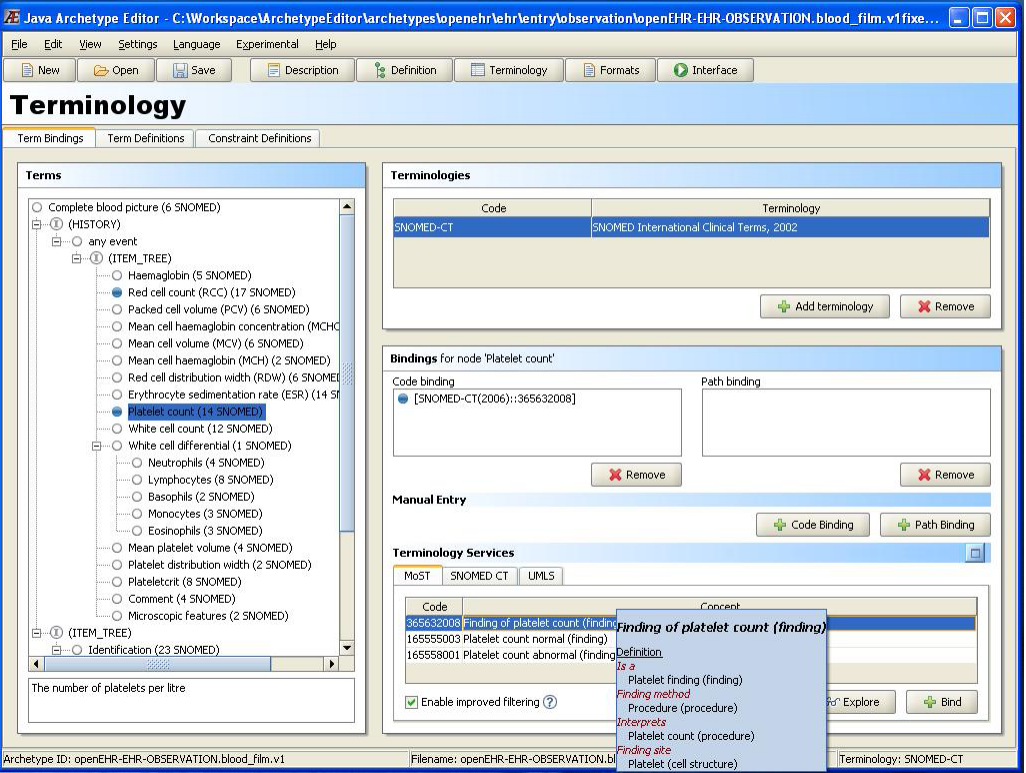
**Terminology view**. The terminology binding view showing bind suggestions extracted from SNOMED CT by MoST. 14 results were returned by MoST for 'platelet count'. The user has reduced the number of results by selecting only the 'finding' and 'procedure' categories of SNOMED CT. The results can then be related to each other and surrounding context using the built in TermViz feature by clicking the 'Explore' button.

The candidate mappings can be viewed in simple tabular form, in Figure 4, in the editor along with the facility to further explore the relevant SNOMED CT hierarchy using the visualization technique described below. See [10] for comprehensive information regarding MoST.

### Terminology visualization

**Figure 5:**
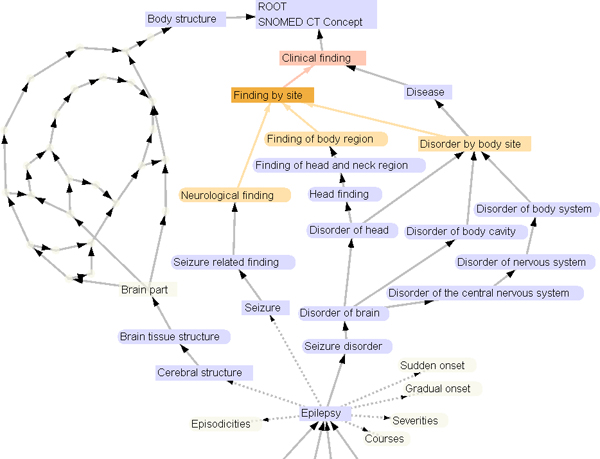
**TermViz**. Visualizing a part of the SNOMED CT hierarchy in TermViz. The graph can be interactively explored and expanded.

Large terminology systems with complex intertwined structure can be hard to navigate and get acquainted with. Free-text queries are possible entries into the exploration of such systems and the way results are presented has impact on the user's ability to grasp the overall structure of the system. Complex hierarchies like the one used in SNOMED CT, where nodes have multiple parents and several other relationship types, makes visualization challenging. A previous paper [3] presented a prototype, called *TermViz*, applying well-known methods from the fields of Information Visualization and Graph Drawing like 'focus+context' and self-organizing layouts. The user can simultaneously focus on several nodes in terminology systems and then use interactive animated graph navigation for further exploration without loosing context. 'Semantic zooming' i.e. reducing the amount of visible information, e.g. text labels far from focused nodes, is also available, see Figure 5. This part of the tool can also be used as a stand alone SNOMED CT browser. Updates regarding TermViz are available at 

## Results

**Figure 6:**
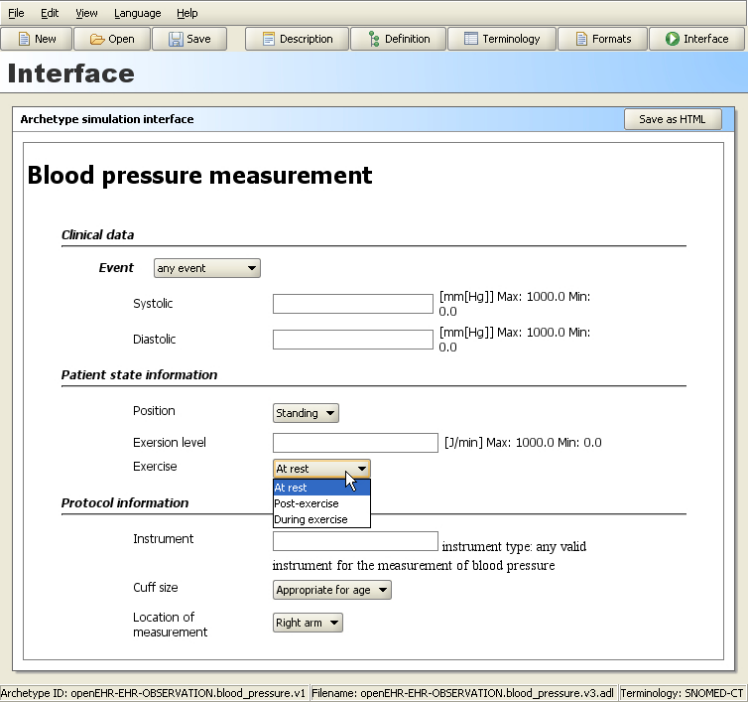
**Interface view**. The interface view of the archetype editor showing how a blood pressure archetype might be rendered in an EHR system.

In this section the integrated application is demonstrated using the blood pressure archetype, shown in the interface view of the editor illustrated in Figure 6

The *definition view of the editor *(see Figure 2) can be used to:

• structure and name the fields in the archetype

• mark fields as mandatory or optional

• restrict format and kind of information to be allowed in a field

In an archetype the 'fields' described above are nodes within a tree structure. Nodes can be bound to terminologies, such as SNOMED CT, as seen in Figure 4. The archetype is sent to the remote MoST-service (accessed using a SOAP-based Web service). In the tree structure to the left are labels ending with e.g. (14 SNOMED) indicating that MoST has found fourteen candidate mappings for the node. Upon selecting a node the suggestions are shown in the list at the bottom right of the screen. The SNOMED CT codes can be selected and 'bound' to the archetype node. A blue dot in front of a node shows that it has been bound to one or more terms in the currently selected terminology. Holding the cursor over a candidate mapping brings up a tool tip (the blue box) showing a short definition of the term.

Free text queries for individual nodes can also be sent to UMLS or to a database containing SNOMED CT tables if locally available.

Results from terminology services can be explored using visualization. On clicking the "Explore" button (Figure 4) an interactive graph opens, as visualized in Figure 5. The graph is constructed by climbing the hierarchy using the IS-A relations starting from the search results ending at the top concept. Other types of relations can also be explored by selecting any node. In addition to exploration, archetype bindings can be created from the graph view as well.

The archetype editor download, and more information can be found at 

## Discussion

Archetype based systems have only been implemented and deployed in limited numbers yet . We believe that semantic interoperability through the archetype approach will have greater chances of success if extensive bindings to terminologies are provided. Finding the right terms to bind is a difficult task but the effort to achieve terminology bindings may be reduced with the help of our methods and tools. The integrated editor eliminates the need for users to swap applications to find appropriate terminology entries. The mapping process is further assisted by the ability to get candidate mappings from MoST.

Visually relating results from the terminology services (instead of only browsing a list) may assist the user in making the correct binding even if there are a large number of terms returned.

### Future work

The term binding problem between two independent models (here the openEHR Reference model and SNOMED CT) and the logical control of post-coordination offer challenging tasks [11]. Post-coordination, i.e. the possibility to combine SNOMED CT concepts from different hierarchies, increases the logical complexity of the problem, e.g. combinations like an observable entity (tumour stage), a body structure (structure of thyroid) and a context-dependent category (family history of). Many coordination variations may in the end mean the same thing, e.g. a post-coordination may be equivalent to an existing pre-coordination or another post-coordination. Logical contradictions also have to be checked for and avoided.

Currently only terminology service assistance for equivalence bindings, i.e. 'this archetype node is synonymous to this SNOMED CT concept' is available in the editor, i.e. 'term bindings' in the archetype formalism [12]. Archetypes also support 'constraint bindings' that in addition to informal text descriptions would allow for more advanced formal bindings to terminologies using compositions of concepts and relations. The formalism for this is not well specified by openEHR as yet, see appendix, but if it becomes expressive enough the archetype editor could:

• assist post-coordination of concepts at the time of archetype creation (e.g. the ones provided by MoST). From the perspective of the clinician using the archetype this could be regarded as a pre-coordination (pre-runtime).

• constrain allowed post-coordinations at runtime, like 'allow any sub-concept of the SNOMED body position concept, but not body position itself' instead of enumerating a list like in Figure 4.

A powerful constraint binding formalism should allow inclusion and exclusion of arbitrary subsets.

The granularity and the degree of compositionality of an archetype also affect the terminology bindings and types of term-coordination possible. See, for example, the difference in the modelling of 'Exercise' (enumerated options) and 'Instrument' (free text) in Figure 6.

Caution is needed if we want to interpret the bindings to do automated reasoning. Formal methods addressing these problems are being researched by one of the authors (Rector). We believe that automated support for formal logical control of terminology bindings and post-coordination in tools like the archetype editor and EHR systems must be added in order to handle the logical complexity described above.

Since the tools discussed in this paper have been developed on the principles of general applicability, it is expected that other terminology systems such as GALEN  or, FMA Foundational Model of Anatomy,  can serve as a second use-case. HL7 V3 models  are quite similar in purpose to archetypes and may also be investigated for demonstrating the mapping methodology.

The integrated editor has been publicly released and is freely available as 'Open Source'. Feedback and future user-based evaluation results can be used for further improvements. How well and easily archetype based clinical models can be mapped to terminology systems is beyond the scope of this paper but such future studies might be helped by this integrated tool. After the initial publication of this paper two of the authors, RQ and AR, have conducted user studies using the integrated editor. This is described in RQs PhD thesis [10] and related papers.

## Appendix

Terminology constraint bindings can in archetypes be stated as 'placeholder constraints' as discussed in detail in chapter 5.3.9 of [12]. These constraints are usually exemplified as URLs intended to point to some future terminology server with a query format yet to be formally specified. By the time of the initial conference publication of this paper some specification documents and example archetypes [13] showed terminology binding URLs similar to http://tqs.openehr.org?terminology_id=SNOMED-CT;has_relation=102002;with_target=57134006 thus containing some terminology related semantics, but as of this writing the preference in specifications and examples seem to be opaque references to IDs of predefined queries like http://tqs.openehr.org/2938495 from [4] or using URL query strings as in [12] producing URLs with endings like ?query_id=12345.

Exactly how, where, when and by whom bindings between archetypes and terminology systems should be created and maintained is currently far from obvious. Trying to capture the moving target of terminology binding for archetypes in a static publication like this paper is impossible. The following list points out related sources of ongoing work and discussion and is intended as starting points for the interested reader:

• NHS work

∘ **The NHS CFH Electronic Health Record Content Technical Advisory Group **wiki page at  is good navigation hub, see presentations and working documents. The working document "**Terminology Binding Requirements and Principles" **by D Markwell found there is a recommended read and partly covers the same things as discussed in [11].

∘ An approach suggesting patterns for externalising code sets and identifiers as separate reusable sub-archetypes is outlined in the draft **NHS CfH Content Model Design Guidelines **

• The openEHR wiki 

∘ E.g. the "Archetypes and Terminology" wikipage 

• The openEHR mailing lists and archives 

∘ E.g. discussions regarding how to fit composite SNOMED CT expressions into archetypes, see 

## Competing interests

The authors declare that they have no competing interests.

## Authors' contributions

All authors had part in conceiving the study, and have substantially contributed in drafting and revising the manuscript.

MF developed the Archetype editor (starting as a master thesis project together with Johan Hjalmarsson) initiated and supervised by ES, MN, HP & HÅ. MF wrote major parts of The Archetype Editor section and implemented major parts of the integration assisted by RQ, ES & MN.

MN wrote major parts of the background, Authors' contributions and References.

DK helped ES update and revise the paper for BMC publication, especially regarding recent terminology binding developments.

RQ developed the the MoST system supervised by AR and wrote major parts of The MoST System section

ES developed TermViz supervised by HÅ & HP. ES was the coordinator and editor of the manuscript and wrote major parts of the sections Teminology visualisation, results, discussion, appendix and the abstract.

All authors read and approved the final manuscript.
